# Current Trends in Ideal Nasal Aesthetics Show Younger Patients Have a Preference Toward Longer Augmented Noses

**DOI:** 10.1093/asjof/ojad069

**Published:** 2023-08-04

**Authors:** Anmol A Patel, Alexandra R Gordon, Alexandra N Townsend, Jinesh Shah, Evan S Garfein, Oren M Tepper

## Abstract

**Background:**

Aesthetic norms fluctuate over time and often result in generational differences in preferred ideal nasal aesthetics. While some traditional concepts of the ideal nasal aesthetic have been suggested in our literature, there has been no study to date that has identified contemporary preferences across different age groups.

**Objectives:**

To understand the general population's current perception of ideal nasal profiles.

**Methods:**

Two-dimensional images of female noses (*n* = 10) of varying ethnicities were simulated to alter either the radix height or nasolabial angle (NLA) independently. Radix height was manipulated by increasing or decreasing the height by 5 mm relative to baseline. For NLA, 3 images were created with the following measurements: (1) 90°, (2) 100°, and (3) 110°. Groups were categorized by generation and age at the time of completing the study: Generation Z (Gen Z; age 18-23), Millennial 20s (age 24-30), Millennial 30s (age 31-39), and Generation X (Gen X; age 40-55). Each figure consisted of either 3 variations in radix height (*n* = 10) or 3 variations in NLA (*n* = 10). Within each figure, volunteers were asked to choose their preferred nose.

**Results:**

The younger generations, Gen Z and Millennial 20s and 30s, preferred a more augmented radix compared to Gen X which preferred a baseline radix height. Gen Z, Millennial 20s, and Gen X preferred a 90° NLA, while Millennial 30s preferred an NLA of 100°.

**Conclusions:**

The authors found that younger populations (Gen Z, Millennial 20s, and Millennial 30s) preferred a more augmented appearance to the nasal radix and, on average, a more acute NLA than published data suggest.

**Level of Evidence: 3:**

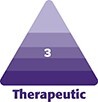

The nose is central to facial aesthetics and, therefore, rhinoplasty can be critical to restoring facial balance and achieving the patient's aesthetic goals. Aesthetic norms may fluctuate over time, and this may result in generational discrepancies regarding nasal aesthetic preferences. While classical ideals have been described, to our knowledge, no study has identified the contemporary ideal nasal profile across various age groups.^1^

Many patients requesting rhinoplasty or nonsurgical rhinoplasty desire minor adjustments to their nose, while others prefer more drastic changes. This wide range of preferences emphasizes the importance of patient involvement in the decision-making process.^2^ Previous studies have shown that while a minority of the population meets aesthetic ideals, these individuals still have subjectively attractive faces.^3,4^ This underscores the point that patient satisfaction ultimately depends on the patient's perception of the result and requires the surgeon to incorporate the patient's aesthetic preferences into their surgical plan. It is known that aesthetic preferences vary across ethnicities, and it is important to consider that neoclassical principles of aesthetic ideals may not be ideal for all cases. The purpose of this study is to delineate the preferences for an attractive nasal profile among various ethnicities and age groups.

## METHODS

### Photography and Simulation

Photographs of female volunteers (*n* = 10) of varying ethnicity and age were taken in profile view with an iPhone 11 Pro (Apple, Inc., Cupertino, CA). Each volunteer's actual dorsal length was measured using calipers to create a ruler within Adobe Photoshop to adjust radix height by 5 mm intervals and nasolabial angle (NLA) by 10° intervals. The NLA was defined as the angle between the base of the columella and the white roll of the upper lip.^5^ Our group has previously used these landmarks to define the NLA and chose to use this definition in our study for consistency.

**Figure 1.**
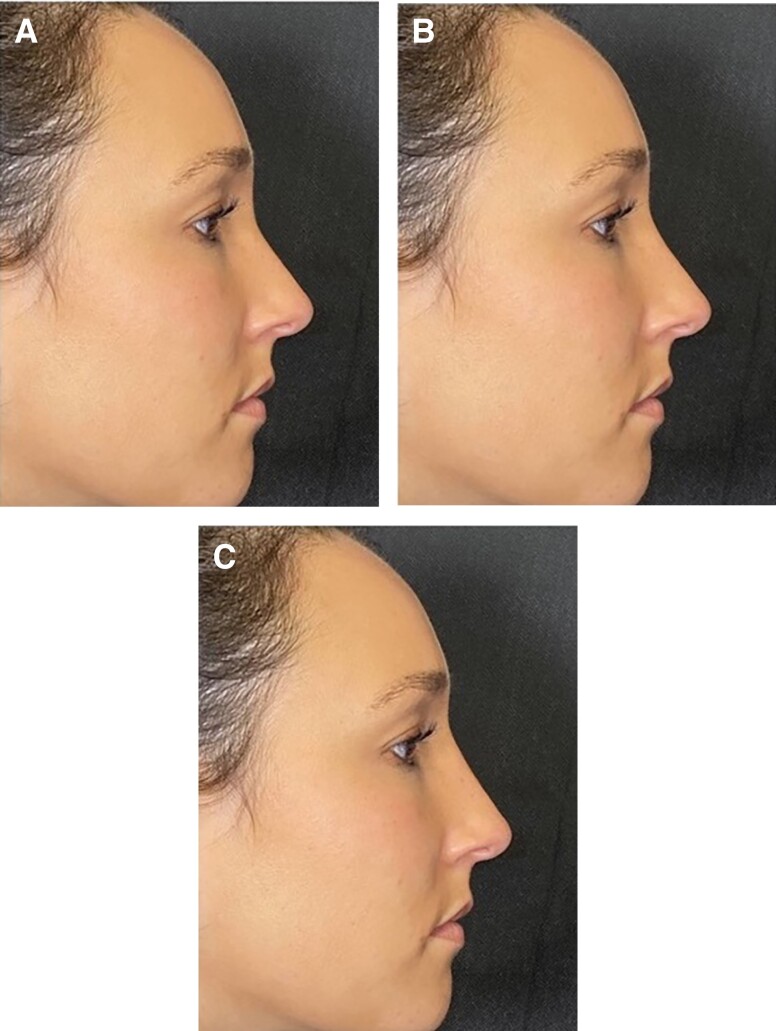
Radix height preference survey. A 32-year-old female's radix height was manipulated in isolation relative to baseline to yield 3 images: (A) decreased 5 mm, (B) baseline, and (C) increased 5 mm.

**Figure 2.**
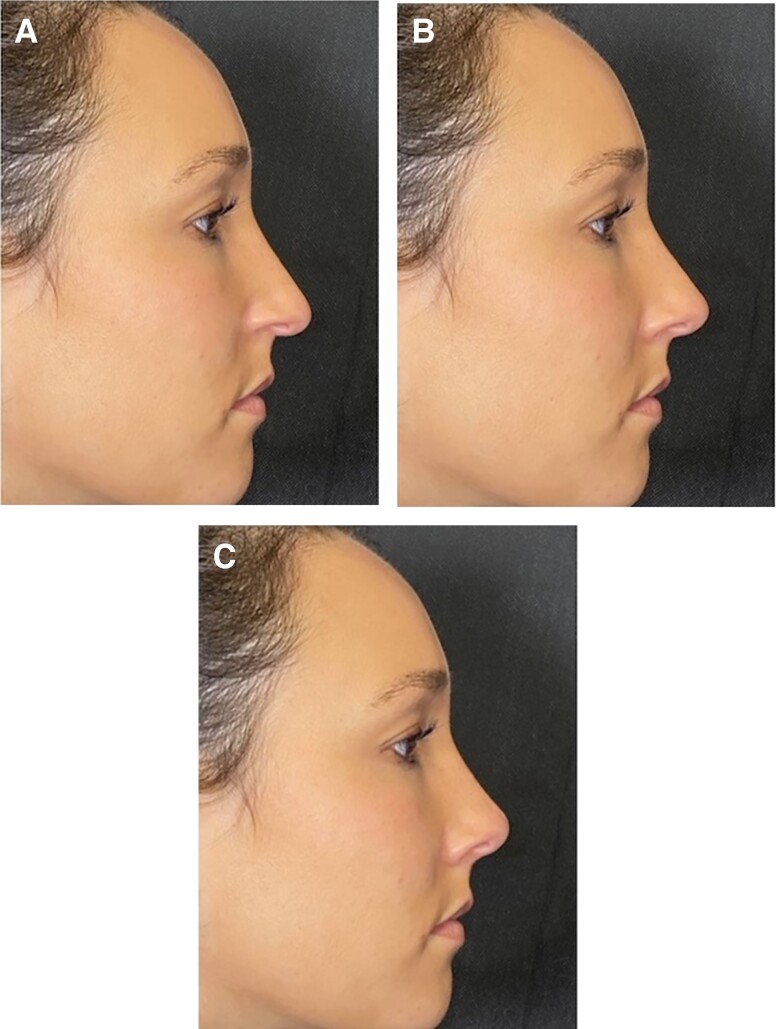
Nasolabial angle (NLA) preference survey. A 32-year-old female's NLA was manipulated in isolation relative to baseline to yield 3 images: (A) 90°, (B) 100°, and (C) 110°.

Using Photoshop CC (Adobe Systems, Inc., San Jose, CA), 10 volunteers’ images were simulated to achieve variations in either radix height or NLA. The isolated alterations in radix height produced 3 images: decreased 5 mm, baseline, and increased 5 mm (Figure 1) and the alterations in NLA produced images representing 90°, 100°, and 110° (Figure 2).

### Study Design

The authors recruited volunteers from the general population over a 2-month period, from January to February 2021. Volunteers were blinded to the purpose of this study. A valid email address was required to ensure no repeat entries were included. In the event that a returned survey was incomplete, the responses were discarded and the survey was excluded from analysis. The single exclusion criterion was a respondent age under 18 years old.

An electronic survey was designed using Qualtrics (Provo, UT). The survey requested demographic data (age group, self-identified race, education level, and profession) and responses to images presented. Volunteers were asked to evaluate a total of 20 figures. Each figure presented an individual's nasal profile with 3 variations in the radix height (5 mm decrease, baseline, 5 mm increase; *n* = 10) or 3 variations in the NLA (90°, 100°, and 100°; *n* = 10). Respondents were asked to select their aesthetic preference for each parameter. The authors have no conflicts of interest to disclose. Written consent was provided, through which the subjects agreed to the use and analysis of their data.

### Statistical Analysis

After completion of data collection, the de-identified data were transferred to a Microsoft Excel document and processed anonymously. Statistical analysis was performed with SPSS Statistics for Windows, version 24.0 (IBM, Armonk, NY). Independent sample *t* tests were performed to compare preferences for radix height and NLA for each volunteer age group subset. Pearson correlation coefficients were calculated to determine correlations between aesthetic preferences and demographics. Statistical significance was determined by the Pearson χ^2^ test, *t* test, and multivariate analysis. A value of *P* < .05 was considered statistically significant. This study was approved by the Albert Einstein College of Medicine Institutional Review Board (no. 2020-12536).

## RESULTS

### Study Demographics

A total of 218 survey responses were recorded, of which 41 (19%) were excluded from this study due to incomplete responses, for a provision rate of 81%. Age groups were categorized by generations: Generation Z (Gen Z) 18 to 23 (16.4%, *n* = 29), Millennial 20s 24 to 30 (44.6%, *n* = 79), Millennial 30s 31 to 39 (22%, *n* = 39), and Generation X (Gen X) 40 to 55 (17%, *n* = 30). The breakdown of patient demographics is shown in Table.

**Table.** Demographic Information

Demographic	*n* (%)
Age	
18-23 (Gen Z)	29 (16.4)
24-30 (Millennial 20s)	79 (44.6)
31-39 (Millennial 30s)	39 (22)
40-55 (Gen X)	30 (17)
Gender	
Male	58 (33)
Female	119 (67)
Ethnicity	
White/Caucasian	80 (45)
Black/African American	5 (3)
Hispanic/Latino	5 (3)
Asian	78 (44)
Hawaiian/Pacific Islander	0 (0)
Native American	0 (0)
Other	9 (5)
Rhinoplasty consideration/had surgery	
Yes	36 (20)
No	141 (80)

### Preferred Radix Height

**Figure 3.**
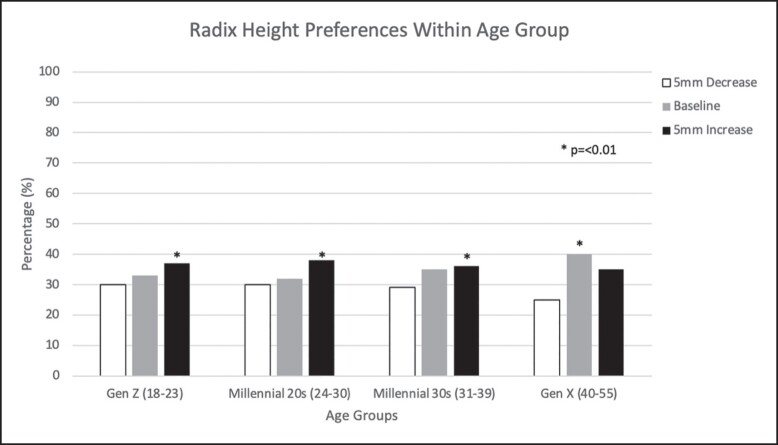
Percentile of radix height preferences in each age group. The *y*-axis shows the percentage of volunteers in each group that chose 1 of 3 choices for radix height. The *x*-axis shows aesthetic preference, and each age group’s preference for radix height.

All age groups on average preferred a radix height larger than baseline (Figure 3). Gen Z, Millennial 20s, and Millennial 30s preferred a 5 mm increased radix height compared to baseline (*P* ≤ .01). Interestingly, Gen X preferred the unaltered, baseline radix position (*P* < .01). For radix height preference, there was no statistically significant difference for race, education level, and personal experience/knowledge of rhinoplasty.

### Preferred Nasolabial Angle (NLA)

**Figure 4.**
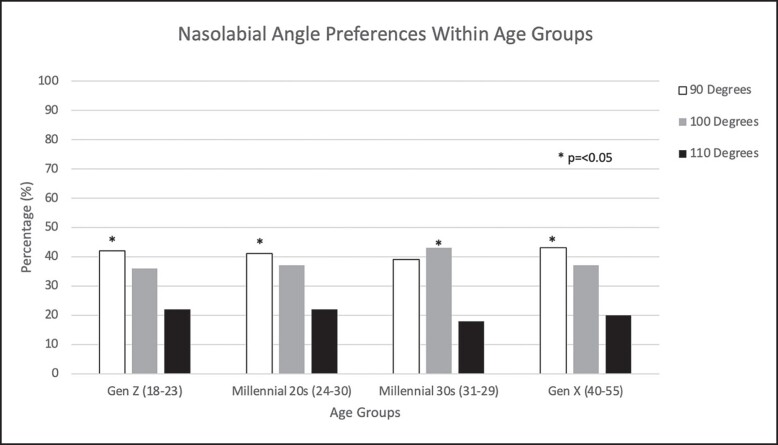
Percentile of NLA preferences in each age group. The *y*-axis shows the percentage of volunteers in each group that chose 1 of 3 choices for the NLA presented as images. The *x*-axis shows aesthetic preference, and each age group’s preference for NLA.

Gen Z and Millennial 20s preferred an NLA of 90° (*P* ≤ .05; Figure 4). Gen X had similar results to the 2 youngest generations and chose an NLA of 90° (*P* ≤ .05). Millennial 30s, however, favored a more obtuse NLA of 100° compared to the 3 other generational groups (*P* = .05).

There was a statistically significant difference between races with Caucasians and Hispanics/Latinos, preferring an intermediate angle of 100°, while African Americans, Asians, and others chose a more acute NLA of 90° (*P* < .001).

## DISCUSSION

The size and shape of the nose are vital to the appearance of the overall face, as the nose is one of the most prominent determinants of facial attractiveness.^6^ Given the subjective nature of aesthetics, it is important for the surgeon to consider the standards of beauty for the patient's demographic. Our group sought to determine and delineate potential differences in perceived attractiveness of the nasal profile across age groups and ethnicities by manipulating radix height and NLA.

A key finding in this study is the preference of a more augmented radix, manifesting visually as a longer nose, in Gen Z, Millennial 20s, and Millennial 30s. Our data suggest that younger generations find longer noses more aesthetically pleasing compared to older generations (Gen X). The preference toward an augmented radix among the younger population may be due to the emergence of the use of filler for nonsurgical rhinoplasty, its increased visibility to the public through social media platforms, and how these minimally invasive procedures increase dorsal height to potentially alter aesthetic norms.^7^ Although the frontonasal angle and maxillary projection may influence the appearance of the radix, these landmarks were not assessed. The preference for a more augmented nose among younger respondents highlights a societal shift and evolution of the standard of beauty through generations, with a focus on a more natural-appearing profile.

Interestingly, the most attractive NLA demonstrated in this study was an average of 98°. This challenges previously published data on ideal nasal aesthetics, which notes the most attractive NLA exists between 100.9° and 108.9° in females.^8^ Our data suggest there is a preference for a more de-rotated tip than previously suggested, with 3 out of 4 study populations choosing the acute NLA option (90°). The preference for a more acute NLA may be in response to the surgically over-rotated nose, commonly referred to as a “ski-slope” or “pig” nose. Interestingly, those with previous personal experience with rhinoplasty, either having had one or considered one, chose the intermediate NLA of 100°, while respondents who did not have previous rhinoplasty exposure chose a more acute 90° angle. This suggests that respondents who have not considered rhinoplasty have a predilection toward a more acute NLA and may have more conservative preferences toward nasal tip rotation than their counterparts.

Although the survey distributed to reviewers requested some demographic data, the survey did not include reviewer gender or geographic location within the United States, which poses a limitation for this study. Unfortunately, we are unable to determine how these factors might have affected his/her choice of the preferred nasal profile. Additionally, our sample size was limited, and the form of survey distribution may have introduced sampling bias through our respondent recruitment. The use of crowdsourcing may have provided a more substantial and diversified sample size. Increased diversity might have allowed our study to expand the number of demographic groupings to confer more information regarding specific nasal profile preferences.

The findings of our study add to the current literature describing the nuances of nasal profile preferences across various demographics.^8,9^ Our results specifically identified the variance of the preferred radix projection and NLA across 4 generations and multiple ethnicities. This added information may aid surgeons in providing more individualized and specific surgical options in the consultation process and beyond to patients based on their demographic.

Our group chose to include volunteers of multiple ethnicities to provide a more diverse cohort to represent the general population. Ethnicity and culture are significant factors to consider in rhinoplasty planning, as standardized definitions of beauty may not align with non-Caucasian patients’ goals.^9^ Sinno et al described significant variations in NLA preferences among certain ethnic groups.^8^ Their group found ethnic groups with more prognathic features, such as West African and Brazilian Black populations, preferred more acute NLAs. Our study supports their findings, as our data demonstrated that respondents who identified themselves as African American had a statistically significant preference for a more acute NLA, along with Asians and others when compared with other ethnic groups.

## CONCLUSIONS

The ideal nasal profile varies among generations and ethnicities. Our group found that younger populations (Gen Z, Millennial 20s, and Millennial 30s) preferred a more augmented appearance to the nasal radix and, on average, a more acute NLA than published data suggest. Our data challenge conventional ideals in nasal aesthetics, suggesting trends toward longer noses and less rotated nasal tips.
